# Medical Team Intervention Into a Long-Term Care Health Facility in Japan During the First Wave of the COVID-19 Pandemic

**DOI:** 10.7759/cureus.61042

**Published:** 2024-05-25

**Authors:** Mayuko Saito, Keiichiro Kita, Ippei Sakamaki, Yoshihiro Yamamoto, Seiji Yamashiro

**Affiliations:** 1 General Internal Medicine, Toyama University Hospital, Toyama, JPN; 2 Infectious Diseases, University of Fukui, Fukui, JPN; 3 Department of Clinical Infectious Diseases, Toyama University Graduate School of Medicine and Pharmaceutical Sciences, Toyama, JPN; 4 Department of Internal Medicine, Asahi General Hospital, Asahi, JPN

**Keywords:** eldery, primary care physician, nursing homes, cluster, covid-19

## Abstract

Nursing homes face a high risk of coronavirus disease 2019 (COVID-19) infection; in the early stages of the pandemic, outbreaks in nursing homes resulted in significant deaths among residents. Our medical team intervened in one nursing home struggling to cope with the COVID-19 pandemic. We analyzed the outcomes of 65 residents (52 women and 13 men; mean age, 89 years) during the first wave of infection, as well as changes in resident and staff numbers after the pandemic subsided. The mortality rates in the early and late transfer groups for the first three months of our intervention were 46.7% and 19.2%, respectively. The number of residents and staff fell to 34 and six, respectively, at their lowest point, but recovered to 64 and 33, respectively, by August 2023. Since the successful containment of the outbreak, no clusters of COVID-19-related illnesses have been observed at the facility despite nine infection waves occurring across Japan. Improving staff precautions, designing facilities with effective zoning, and sharing information with government agencies are essential for preventing healthcare-associated infections. Hence, an inter-professional team approach is important to support residents, and ongoing mental health support for staff is essential to maintain optimal healthcare quality in nursing home facilities.

## Introduction

The COVID-19 pandemic has severely impacted care facilities for older adults. As of April 27, 2023, the number of reported cluster cases in nursing homes in Japan, including facilities with multiple occurrences, has reached 27,336 [1]. Nursing home residents are generally at a high risk of infection due to individual factors such as age, pre-existing comorbidities, and care needs; as well as facility factors such as a closed system and close care between patients and staff. These factors played crucial roles COVID-19 pandemic as well [2-4]. Older individuals also have a higher rate of severe COVID-19 manifestations and mortality [5], as observed particularly during the early phase of the pandemic [6]. Therefore, controlling the pandemic in its early stages was crucial for preventing mortality in older residents of long-term care facilities [7-9].

During the first wave of COVID-19 infections in 2020, a Japanese nursing home (Toyama Rehabilitation Home, Toyoma, Japan) struggled to cope with the rapid rise in patients infected with the disease at their facility. Moreover, exhausted caregivers left the facility to seek other employment, making it challenging for the remaining staff to maintain daily care. In April 2020, our medical team intervened to help control the spread of the infection in Facility A, which was in disarray due to the COVID-19 first wave, among other facilities facing similar issues. Since then, we have continued to work with the facility to implement infection control measures and monitor the progress of post-wave outbreaks. There have been several reports of medical teams intervening in such care facilities [10-12]. However, there are currently no reports in the literature regarding continued intervention in these facilities after major COVID-19 infection waves. Therefore, in this study, we retrospectively validated the effectiveness of our interventions following control of the COVID-19 epidemic in Japan.

## Materials and methods

We analyzed the outcomes of 65 residents (52 women, 13 men; average age, 89 years) and 64 facility staff members after our initial intervention for the first wave of COVID-19 infections in Japan (13th-20th week, 2020). The evaluation period spanned from April 1 to June 30, 2020.

We used the SARS-CoV-2 real-time reverse transcription polymerase chain reaction (rRT-PCR) test to make all definitive diagnoses of COVID-19. Clinical onset of COVID-19 infection was defined as a fever of over 37.5°C, respiratory symptoms, and arthralgia, in accordance with the Japanese Ministry of Health, Labor, and Welfare’s guidelines [13]. Based on our experience, appetite loss was also included as an initial symptom. COVID-19-related deaths were defined as any deaths accompanied by positive PCR test results.

COVID-19-infected residents were divided into early and late transfer groups for statistical analysis, and the time from diagnosis by PCR to hospital transfer in 28 days was used as the cutoff date. Fisher’s exact test, performed using SPSS software, version 28 (IBM Corp., Armonk, NY, USA), was used to compare the two groups. Statistical significance was set at p < 0.05 (two-tailed value). This was a small-sample observational study; therefore, we did not calculate the necessary sample size or perform multivariate regression analysis. The Ethical Review Board of Toyama Hospital approved this study (approval number: R2023015), and the participants had indirect opt-out opportunities through announcements on the website of Toyama Rehabilitation Home, Toyoma, Japan, as an acceptable method of obtaining consent.

Overview of the medical intervention

Toyama Rehabilitation Home is a long-term care health facility in a Japanese city. Before the COVID-19 pandemic began, this facility had 65 residents and 64 staff members. The first COVID-19 case in the prefecture was identified on March 30, 2020. On April 3, one resident at the facility (Case X) developed a fever and was transported to a city hospital for pneumonia on April 16. The following day, she was diagnosed with COVID-19 via PCR testing. This was the first COVID-19 case in the facility. At the time of diagnosis, 20 other residents had developed fevers. Therefore, the city public health center (PHC) conducted PCR tests on all of the residents and staff until April 23 and verified that 63% (41/65) of the residents and 28% (18/64) of the staff were PCR-positive. The PHC confirmed that Toyama Rehabilitation Home had the first case of a COVID-19 infection cluster in a nursing home within the prefecture.

On April 25, the authors (MS and SY), who were certified primary care physicians, started medical interventions in the facility in response to medical support requests from the prefecture. When we first visited the facility, 51 residents remained, and febrile patients were not isolated. The number of staff members had decreased from 64 to six, as a result of hospitalizations to treat COVID-19 and staff leaving over fears of contracting the infection. Waste disposal, cleaning, and kitchen services were temporarily closed to prevent the spread of the infection. Thus, when the intervention was initiated, sanitation and quality of care at the facility were critical. COVID-19 soon spread further throughout the prefecture, making it difficult to transport patients to designated hospitals.

First, we organized a temporary medical intervention team comprising two physicians, one facility doctor, two nurses, two caregivers, and one social worker. As an initial intervention, we gathered information from the residents and shared it with the team. Following this, we rearranged the facility layout, including zoning of infected residents, and referenced the medical guidance released by the Japanese Ministry of Health, Labour and Welfare [13].

As a medical intervention, patients were triaged into two groups: those who were indicated for intensive care and were transported to the hospital immediately and those who could be left in place under observation. Given their clinical conditions and social backgrounds, we cautiously decided to provide end-of-life care to some residents who were unable to express their wishes owing to dementia. We performed daily rounds and administered oxygen, intravenous fluids, and antibiotics to residents in need with the limited medical resources available to us. As rapid testing kits for SARS-CoV-2 were not available at that time, the residents’ clinical conditions were evaluated based solely on vital signs and physical examination findings. Due to staff shortages, we also performed nursing care tasks such as assisting with bathing and meals.

As an external negotiation, we cooperated and shared information with the city Prefectural Countermeasures Headquarters (PCH), the department of clinical infectious diseases of the university hospital, and other designated COVID-19 hospitals. Nurses and caregivers were recruited from all over the prefecture and surrounding regions via these organizations. We also communicated with the residents’ families regularly via telephone and informed them of the conditions of their family members.

## Results

**Figure 1:**
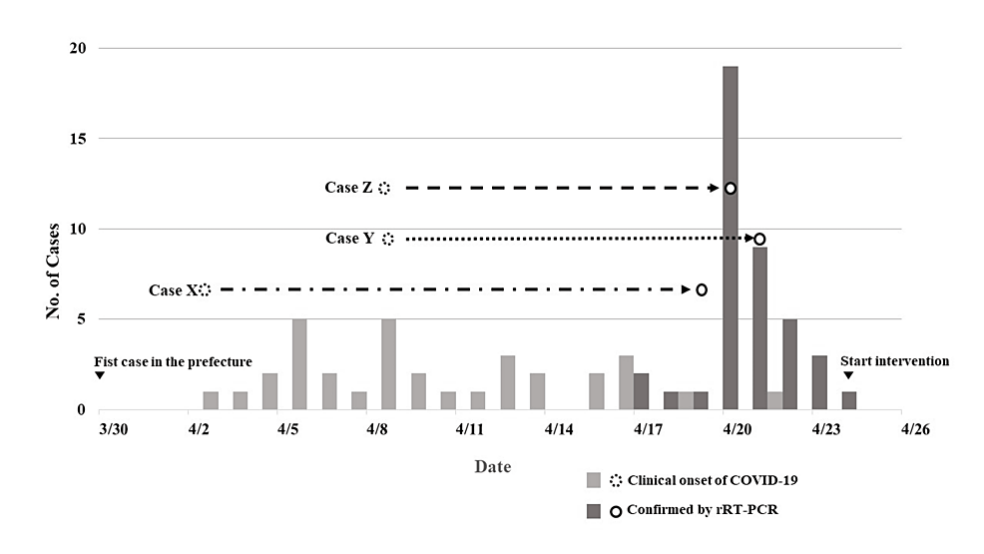
Number of residents infected with COVID-19 during the first cluster outbreak COVID-19: Coronavirus disease 2019; rRT-PCR: Real-time reverse transcription polymerase chain reaction

Figure 1 shows the number of COVID-19-infected residents during the outbreak of the first infection cluster. The first case (case X) exhibited clinical onset on April 3, and the patient was immediately transported for suspected bacterial pneumonia. The second and third transported patients (Y and Z) were initially treated for urinary tract infections and aspiration pneumonia. At that time, there was no outbreak in the prefecture, and COVID-19 was diagnosed after > 10 days.

Table 1 shows the characteristics of COVID-19-infected patients at the facility. The majority of patients were older women with chronic underlying medical conditions. All patients were certified as requiring nursing care, and one-third had very high or extremely high levels of care needs (4 or 5 on a 5-point scale).

**Table 1:** Characteristics of study facility residents with confirmed COVID-19 infections COVID-19: Coronavirus disease 2019, Study facility: Toyama Rehabilitation Home, Toyoma, Japan.

Characteristics	Residents (n = 41)
Median age (range) yrs	88.8 ±7.0 (73–103)
Sex no. (%)	N/A
Male	8 (19.5)
Female	33 (80.5)
Chronic underlying conditions no. (%)	N/A
Hypertension	26 (63.4)
Cerebral vascular disease	14 (34.1)
Cardiac disease	8 (19.5)
Diabetes mellitus	6 (14.6)
Renal disease	1 (2.4)
Pulmonary disease	2 (4.9)
Median level of care needed (range)	3.1 (1–5)

The mortality rate of COVID-19 infection increased with age (Table 2).

**Table 2:** Number of PCR-positive residents and mortality by age rRT-PCR: Real-time reverse transcription polymerase chain reaction

Age range	rRT-PCR positive no.	Deaths no., (%)
70–79	5	1 (20)
80–89	17	4 (23.5)
90+	19	7 (36.8)
Total	41	12 (29.3)

Table 3 shows the outcomes of residents who were transferred to the hospital with confirmed COVID-19. The remaining residents who were not transferred during the observation period were included in the late transfer group. Mortality was higher in the early transfer group than in the late one, although the difference was not statistically significant (p = 0.0834).

**Table 3:** Outcomes of hospital-transferred residents with confirmed COVID-19 COVID-19: Coronavirus disease 2019

	Early transfer group (n = 15)	Late transfer group (n = 26)	Total
Alive	8	21	29
Deaths	7	5	12
Mortality (%)	46.7	19.2	29.3

After approximately one month, all of the residents tested negative for PCR twice. The number of residents decreased to 34 at its lowest point, but recovered to 64 by August 2023. Although the majority of original staff members resigned, new staff were hired, bringing the total to 33. Currently, the facility continues to hold regular infection control meetings to share information on infection and to practice standard precautions. Since the beginning of the pandemic, there have been no clusters of deaths due to COVID-19 at this facility, even though nine major waves of infection have occurred in Japan.

## Discussion

Our medical team had the opportunity to intervene during the early phase of a COVID-19 cluster outbreak at a long-term care facility in Japan. As far as possible, we implemented infection control measures and reduced unexpected infection-related deaths in the facility as much as possible. Further, we provided medical and nursing services with limited staff during the pandemic, and established infection control meetings. The numbers of residents and staff at the facility are now close to pre-pandemic levels.

Previous articles on nursing home interventions during the COVID-19 pandemic have reported that medical teams had to provide medical care only within their facilities despite the various difficulties they faced [10-12]. Moreover, some studies have emphasized the importance of structural control measures during the pandemic [13,14]. Referring to the literature, we discuss the countermeasures against infectious disease pandemics in long-term care facilities, dividing them into three phases: 1) preventing mass infection, 2) outbreak response, and 3) recovery from outbreaks.

Countermeasures against infectious disease pandemics in long-term care facilities

1) Preventing Mass Infection

Nursing homes face a high risk of outbreaks because most residents are easily infected and live in close quarters [15]. Moreover, these facilities generally have inadequate infection control measures and lower COVID-19 testing rates compared to medical facilities [16,17]. Improving staff infection prevention skills, designing facilities with effective zoning, and sharing information with government agencies and medical staff teams are essential to overcoming these limitations [18].

2) Outbreak Response

Pandemics and other disasters pose similar challenges to both healthcare and medical services [19]. Therefore, management of the COVID-19 pandemic should be based on natural disaster preparedness measures. As a specific problem in nursing homes, the interruption of routine care services may increase deaths in non-infected patients. Although routine care is effective in preventing delirium and other medical complications [20], caregivers have traditionally distanced themselves from medical issues. Through the experience detailed in this report, we found that the practical experience of caregivers who are in close contact with older residents daily can be useful in more effectively enhancing medical support. Hence, medical staff should work closely with non-medical staff across professional boundaries to support residents, particularly during pandemic situations.

3) Recovery From Outbreaks

The majority of staff at Toyama Rehabilitation Home, mainly the caregivers, resigned during the first wave of the pandemic. The critical situation caused by the COVID-19 outbreak in the facility led to the clinical staff facing physical and emotional exhaustion [21]. Moreover, inadequate knowledge surrounding infectious diseases can easily lead to prejudice and vilification among nursing home staff. Therefore, ongoing long-term psychological support for staff is essential to maintaining a high quality of care in such facilities.

Limitations

This observational study had some limitations worth noting. As the sample size was small and came from a single nursing home, the study design may have introduced some overall bias. Therefore, our results should be interpreted with caution to avoid overgeneralization. Further research is warranted to explore the usefulness of medical teams led by primary care physicians in nursing homes to cope with pandemic situations.

## Conclusions

The COVID-19 outbreak has severely impacted nursing homes, revealing their vulnerability and inadequate countermeasures against infectious outbreaks. Our experience suggests that medical support teams led by primary care physicians may be able to provide appropriate care within such facilities while also maintaining the local medical management system. Moreover, the intervention may contribute not only to outbreak response but also to the prevention and recovery from outbreaks.
